# Characterization of Insufficiency Fracture and Bone Metastasis After Radiotherapy in Patients With Cervical Cancer Detected by Bone Scan: Role of Magnetic Resonance Imaging

**DOI:** 10.3389/fonc.2019.00183

**Published:** 2019-03-28

**Authors:** Xi Zhong, Jiansheng Li, Linqi Zhang, Binggui Lu, Jinxue Yin, Zhijun Chen, Jian Zhang, Rijie Tang

**Affiliations:** ^1^Department of Radiology, Affiliated Cancer Hospital and Institute of Guangzhou Medical University, Guangzhou, China; ^2^Department of Nuclear Medicine, Affiliated Cancer Hospital and Institute of Guangzhou Medical University, Guangzhou, China; ^3^Department of Radiation Oncology, Affiliated Cancer Hospital and Institute of Guangzhou Medical University, Guangzhou, China

**Keywords:** insufficiency fractures, magnetic resonance imaging, bone scan, cervical cancer, radiotherapy

## Abstract

**Background:** Insufficiency fracture (IF) can show increased uptake on a bone scan (BS). IFs are often misinterpreted as bone metastases if the characteristic “Honda sign” (H-sign) is invisible. The purpose of the present study was to evaluate the utility of magnetic resonance imaging (MRI) alone for the characterization of IF and bone metastasis after radiotherapy in patients with cervical cancer detected by BS.

**Materials and Methods:** Our study included 40 patients with cervical cancer after radiotherapy that showed pelvic emerging increased uptake on a BS during follow-up. Then further MRI examination was performed in all patients. Two radiologists independently reviewed the MR images, and the sensitivity, specificity and accuracy were calculated based on the mean scores. Diagnostic validity of the inter-observer was calculated by using kappa statistics. The gold standard was based on radiologic findings, clinical data and follow-up at least 12 months.

**Results:** A total of 57 emerging bone lesions detected at BS were identified in the reference standard, including 43 IFs and 14 bone metastases. Only 20 patients showed a “H-sign” on the BS images. Using MRI analysis, all lesions detected by BS were found in MRI by both radiologists. On average, the sensitivity, specificity, and accuracy for distinguishing IFs from bone metastases were 95.3% (41/43), 92.8% (13/14), and 94.7% (54/57), respectively. The inter-observer variability was determined to be very good (kappa value = 0.962).

**Conclusions:** MRI is a reliable diagnostic technique for the further characterization of emerging lesions detected by BS, MRI shows great diagnostic efficiency in the differentiation of IF and bone metastasis.

## Introduction

Insufficiency fracture (IF) represents a special category of stress fractures, which most frequently occurs in the pelvis, followed by the proximal femur and the vertebra (1). Postmenopausal osteoporosis is most commonly associated with IF, and other risk factors include the long-term use of bisphosphonate or steroid, rheumatoid arthritis, and hyperparathyroidism (2, 3). Radiotherapy (RT) that is used to detect malignancy is considered a significant cause of IF (4, 5). Several clinical studies have revealed that RT-induced IF is frequent, and reported the cumulative incidences of post-radiation IF at 2 and 5 years were 10–37 and 8–45.2%, respectively (6–11).

IF usually shows increased uptake on bone scan (BS), and the “Honda sign or its variation” (H-sign) is well-known as a characteristic sign of a sacral IF (12, 13). However, the “H-sign” is not always present. Furthermore, it is difficult to differentiate between benign and malignant disease when a patient with a history of malignancy exhibits a H-like pattern or atypical “H-sign.” IFs are frequently occult, and in particular, sacral IFs are usually incorrect diagnoses (9, 10). This kind of incorrect diagnosis may result in unnecessary biopsy and aggressive radio-chemotherapy. Biopsy of a lesion is not recommended due to the high probability of fracture or hemorrhage and the low diagnostic performance (14). Differentiation of RT-induced IF from bone metastasis by using medical imaging is becoming very important for patients with a history of malignancy.

Magnetic resonance imaging (MRI) has the ability to detect fracture lines, and it is also very useful for detecting the soft-tissue component, which is helpful for distinguish IF from malignant disease. MRI is highly sensitive for revealing the reactive bone marrow changes associated with IF during follow-up after RT (15). Recent studies show that MRI is a superior diagnostic tool for occult pelvic IFs (11, 16). As for the differentiation of IFs and bone metastases, a previous study showed that SPE/CT had potential diagnostic value in the characterization of IFs and bone metastases after RT detected by BS (17). Although some studies has assessed the MRI features of IF after RT (9, 11, 15). To our best knowledge, the performance of MRI in the identification of emerging pelvic lesions after RT detected by BS is still unclear. Thus, in our study, we aimed to evaluate the diagnostic utility of MRI for differentiating IFs and bone metastases after radiotherapy in patients with cervical cancer detected by BS.

## Materials and Methods

### Patient Samples

The present retrospective study was approved by our institutional review board. We conducted a retrospective study of 40 patients with cervical cancer between January 2014 and December 2017; all patients received radiotherapy. The subject inclusion criteria included: (1) pathology-proven cervical cancer that received pelvic radiation therapy, (2) pre-treatment BS where available, (3) underwent follow-up BS for excluding bone metastasis after radiotherapy, and showed emerging areas of pelvic abnormal radiotracer uptake, (4) MRI was performed for further diagnosis after pelvic abnormal radiotracer uptake detected by BS, (5) one or more pelvic CT or MRI examinations during the 12 month (at least) follow-up after undergoing MRI. Exclusion criteria included: (1) pelvic metastasis before radiation therapy, (2) pelvic fracture or bone marrow edema before radiotherapy, (3) a history of pelvic trauma.

### Patient Characteristics and Treatments

The mean age was 64 years old (age range 48–78 years old). The mean body mass index (BMI) was 22.5 ± 8.4 (range 14.8–30.5). A total of 32 patients had low back pain (or hip pain) and eight patients were asymptomatic. There was postmenopausal documented history in 29 patients and premenopausal in 11 patients. Among these patients, 35 cases showed cervical squamous cell carcinoma, 4 cases had adenocarcinoma and one showed adenosquamous cell carcinoma according to histopathology. Eight cases were stage IB disease, 22 cases were stage II disease (*n* = 10, IIA; *n* = 12, IIB), eight cases were stage III disease (*n* = 3, IIA; *n* = 5, IIB) and two cases were stage IVA disease based on the International Federation of Gynecology and Obstetrics (FIGO) staging system.

Among the 40 subjects, 30 patients (75%) received definitive radiotherapy, which included a combination of external beam radiation therapy (EBRT) and high-dose-rate intracavitary brachytherapy (HDR-ICBT), concurrent with (*n* = 26) or without (*n* = 4) cisplatinum-based adjuvant chemotherapy. EBRT was prescribed with a median prescribed dose of 50.4 Gy (range, 30.6–66.6 Gy) in 1.8-Gy fractions for 5 days each week using 10- or 15-MV x-rays, followed by 24 Gy at 6 Gy per fraction of HDR-ICBT to point A. Ten patients (25%) received postoperative radiotherapy after surgery, which included a 50.4 Gy at 1.8 Gy per fraction of EBRT for the entire pelvic field; a 4-field box technique was used, followed by 24 Gy at 6 Gy per fraction of HDR-ICBT to point A. In three patients with positive para-aortic nodes, the RT fields were extended up to the levels in which the involved lymph nodes were adequately included. Therapeutic regimens of concurrent chemotherapy consisted of weekly cisplatin (40 mg/m^2^) or weekly nedaplatin (30 mg/m^2^) for 5–6 cycles. Patient characteristics and treatments are summarized in Table 1.

**Table 1:** Patient characteristics.

**Characteristics**	**Value**
*n*	40
Median age (rang)	64 (48–78) years
Median BMI (range)	22.5 (14.8–30.5)
**MENOPAUSAL STATUS**	
Post-menopausal	11
Pre-menopausal	29
**STAGE (FIGO)**	
IB	8
IIA	10
IIB	12
IIIA	3
IIIB	5
IVA	2
**HISTOPATHOLOGY**	
Squamous cell carcinoma	35
Adenocarcinoma	4
Adenosquamous cell carcinoma	1

*BMI, Body Mass Index; FIGO, International, Federation of Gynecology and Obstetrics*.

### MRI Scanning

MRI examinations of the pelvis were obtained using a 1.5 T MR-scanner (Philips Achieva, Philips Healthcare, Best, The Netherlands) with a multichannel receive only surface coil. The imaging protocol comprised an axial T1-weighted spin-echo image [TR/TE, 496/10 ms; matrix, 304 × 332; number of excitations (NEX), 2], axial T2-weighted spin-echo image (TR/TE, 2528/100 ms; matrix, 304 × 325; NEX, 2) and a coronal spectral attenuated inversion-recovery (SPAIR) T2-weighted image (TR/TE, 2400/80 ms; SPAIR TR, 266 ms; matrix, 280 × 306; NEX, 2). Contrast-enhanced axial and sagittal T1-weighted images were obtained in all patients using a SPAIR T1-weighted sequence. The field of view was 22–26 cm, and a section thickness of 5 mm with a 2 mm interscan gap was used for all scanning sequences.

### Bone Scan

The whole-body BS images were obtained 3 h after intravenous injection of 15–25 mCi 99 mTc-MDP, using a dual-head gamma camera equipped with low-middle-energy general-purpose collimators (Symbia T6, Siemens Medical Solutions). In the whole body scan, both anterior and posterior images were acquired in a matrix of 256 × 1,024 and scanning speed of 15 cm/min, and the acquisition energy window was 140-keV (±7.5%).

### Imaging Analysis

Only the lesions in the L5 vertebra, proximal femur and pelvis were included in the analysis, although every patient had further MRI in this area.

For BS analysis, the images were independently interpreted by an experienced nuclear medicine physician (L.Q.Z, with 5 years of experience in nuclear medicine). The emerging locations of increased uptake and “H-sign” were recorded. The “H-sign” is an H-shaped increase in areas of abnormal radiotracer uptake on the sacral body and both alae (12). The variation was an incomplete “H-sign,” which means that part of the letter “H” was invisible (17).

For MRI analysis, two radiologists (B.G.L., J.S.L) with 5 and 10 years of experience in skeleton imaging, respectively, separately and independently reviewed all MR images. The radiologists were informed of pelvic radiation therapy history and the exact locations of increased uptake in BS but they were all blinded to the BS images and diagnostic results, and the radiologists were blinded to patients' clinical symptoms, radiation dose, laboratory results, findings of other imaging modalities, and final diagnosis. The absence or presence of a bone marrow edema pattern and fracture lines was documented for each lesion. A fracture line was defined as a linear structure visualized on either fat-saturated T2-weighted, T1-weighted, or enhanced T1-weighted images. Reactive bone marrow change associated with insufficiency fracture was noted by low signal intensity on T1-weighted images and high signal intensity on fat-saturated T2-weighted images. Each observer recorded the possibility of IF for each lesion by using the following four-point confidence rating scale: a score of 1, not IF; a score of 2, possibly IF; a score of 3, most likely IF; and a score of 4, definite IF. A score of 0 was determined when an observer did not find the lesion in MRI but it was detected by BS. The criteria (9, 11) for diagnosis of IF on MRI were as follows: (a) a lesion shows bone marrow edema pattern with fracture line but without soft tissue mass; (b) a lesion only shows a fracture line without soft tissues mass. When an observer estimated the lesion as a score of 3 or 4, the lesion was classified as positive.

### Reference Standard

The definition of a standard of reference is challenging for a study in which no surgical procedures or pathologic examinations are standardly performed. As mentioned in previous studies, the reference standard was based on all available radiologic findings (BS, CT, or MRI), clinical data and follow-up for at least 12 months, which was then used as a gold standard for our study (11, 16, 17). None of the patients had a biopsy of the bone lesion due to the high probability of fracture or hemorrhage and the low diagnostic performance (14).

### Statistical Analysis

All statistical tests were performed using the SPSS Statistics 16.0 (SPSS Inc., Chicago, IL, USA) software package, and statistical significance was accepted for *P* < 0.05. Categorical data were expressed as numbers and frequency (%), and continuous data were expressed as means and standard deviations. The sensitivity, specificity, and accuracy for diagnosing IF were calculated based on the recorded scores.

Diagnostic validity of the interobserver was calculated by using kappa statistics. A classification of validity on the basis of the k-values was introduced as follows (18): very good (1 ≥*k* > 0.8); good (0.8 ≥ *k* > 0.6); moderate (0.6 ≥ *k* >0.4); fair (0.4≥*k* > 0.2); or poor (0.2 ≥ *k* > 0).

## Result

### Patient-Wise Analysis

**Figure 1:**
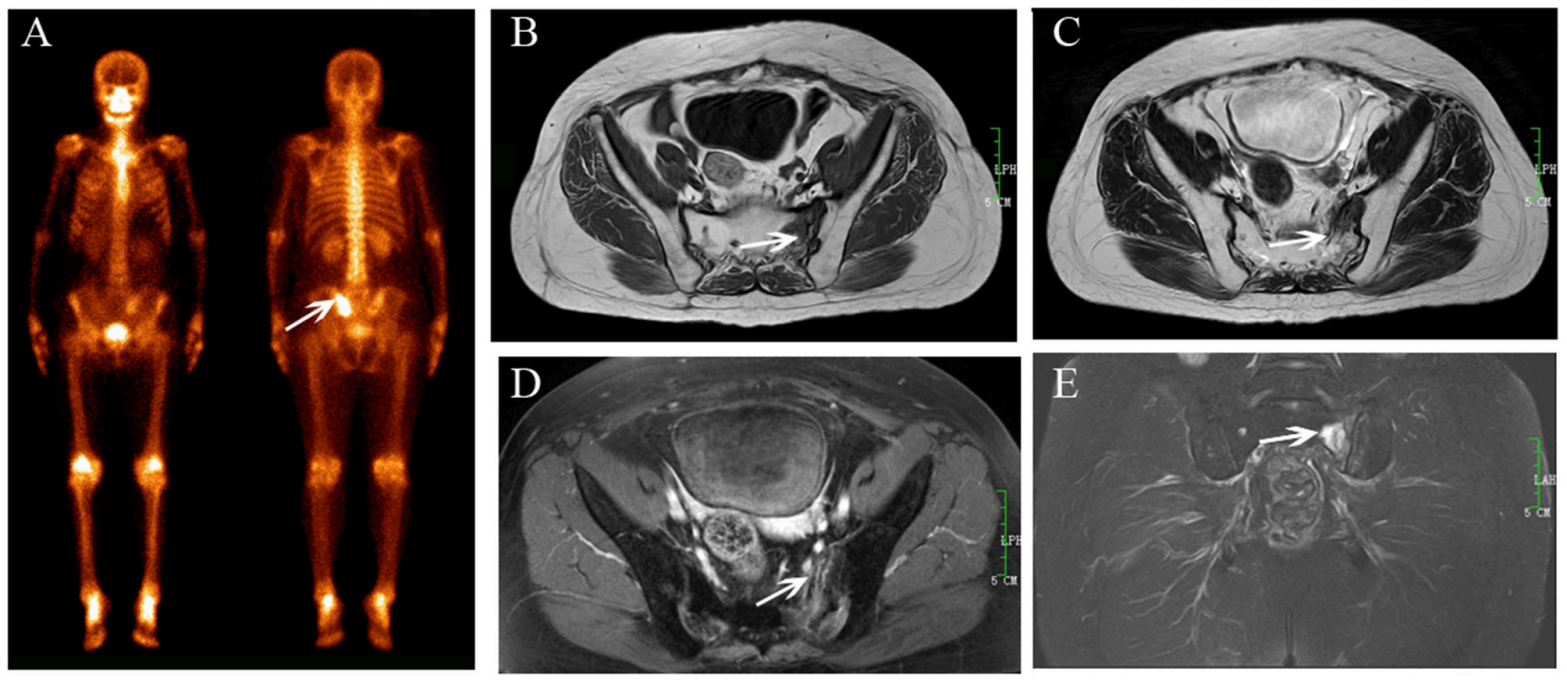
Sacral IF after radiotherapy. Bone scan (BS) **(A)** shows left sacrum with increase uptake similar to a variant of “Honda sign.” The lesion shows hypointensity on axial T1-weighted **(B)**, hyperintensity on both axial T2-weighted **(C)**, and enhanced T1-weighted images **(D)**, and hyperintensity on coronal FS T2-weighted image **(E)**. Fracture line is shown on all MR sequences (white arrows).

**Figure 2:**
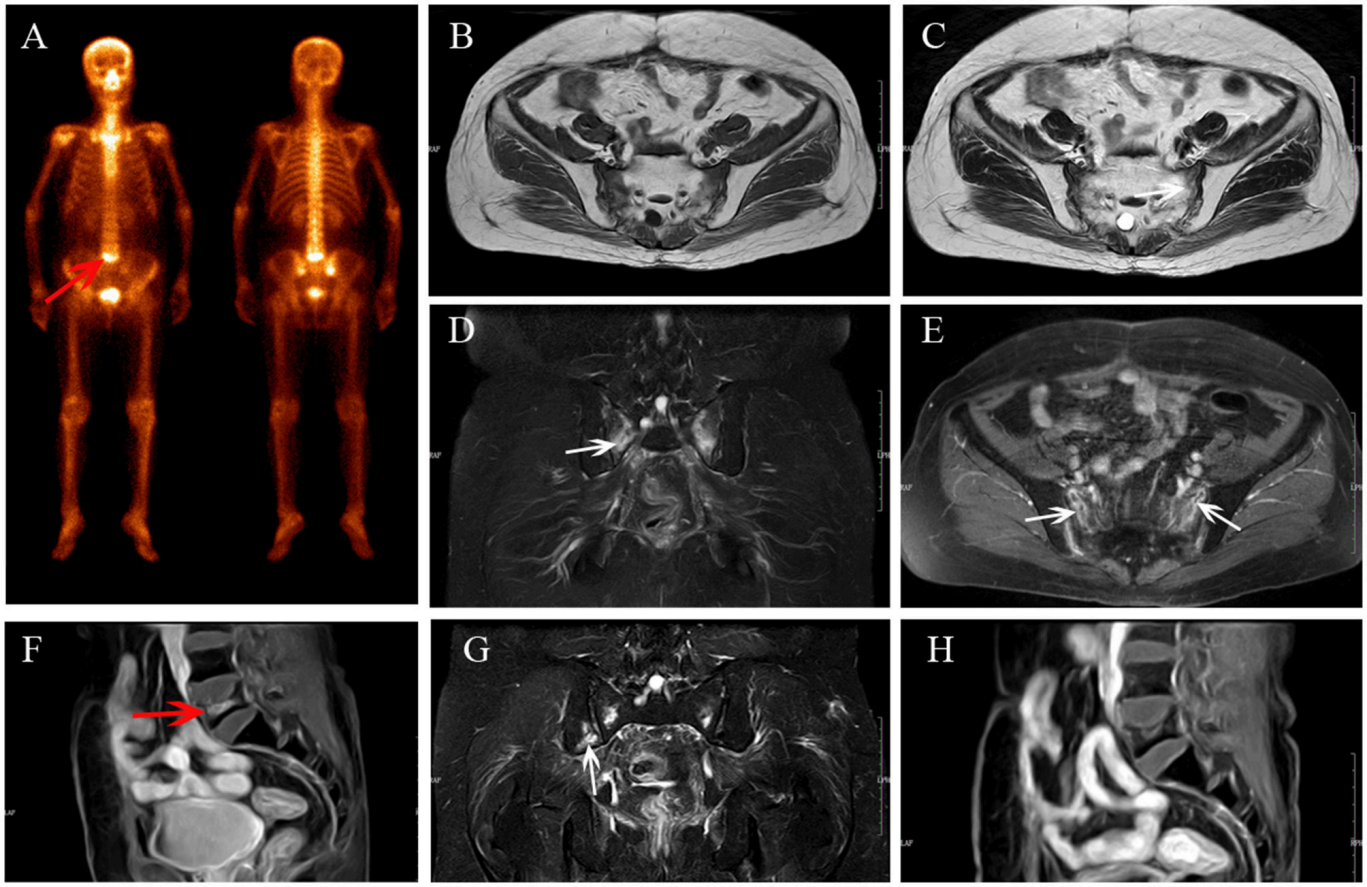
Multiple IFs after radiotherapy. BS **(A)** shows “Honda sign” and an increase in uptake in L5 (red arrow). The bilateral sacrum shows reactive bone marrow change on axial T1-weighted **(B)**, axial T2-weighted **(C)**, and coronal FS T2-weighted **(D)** images, and enhancement on enhanced T1-weighted image **(E)**; fracture line is shown (white arrows). L5 shows compression fracture with enhancement on sagittal enhanced T1-weighted image **(F)**. At MRI follow-up after 14 months, the area of IFs has decreased **(G,H)** and a new fracture occurred in the right iliac (**G**, white arrow).

**Figure 3:**
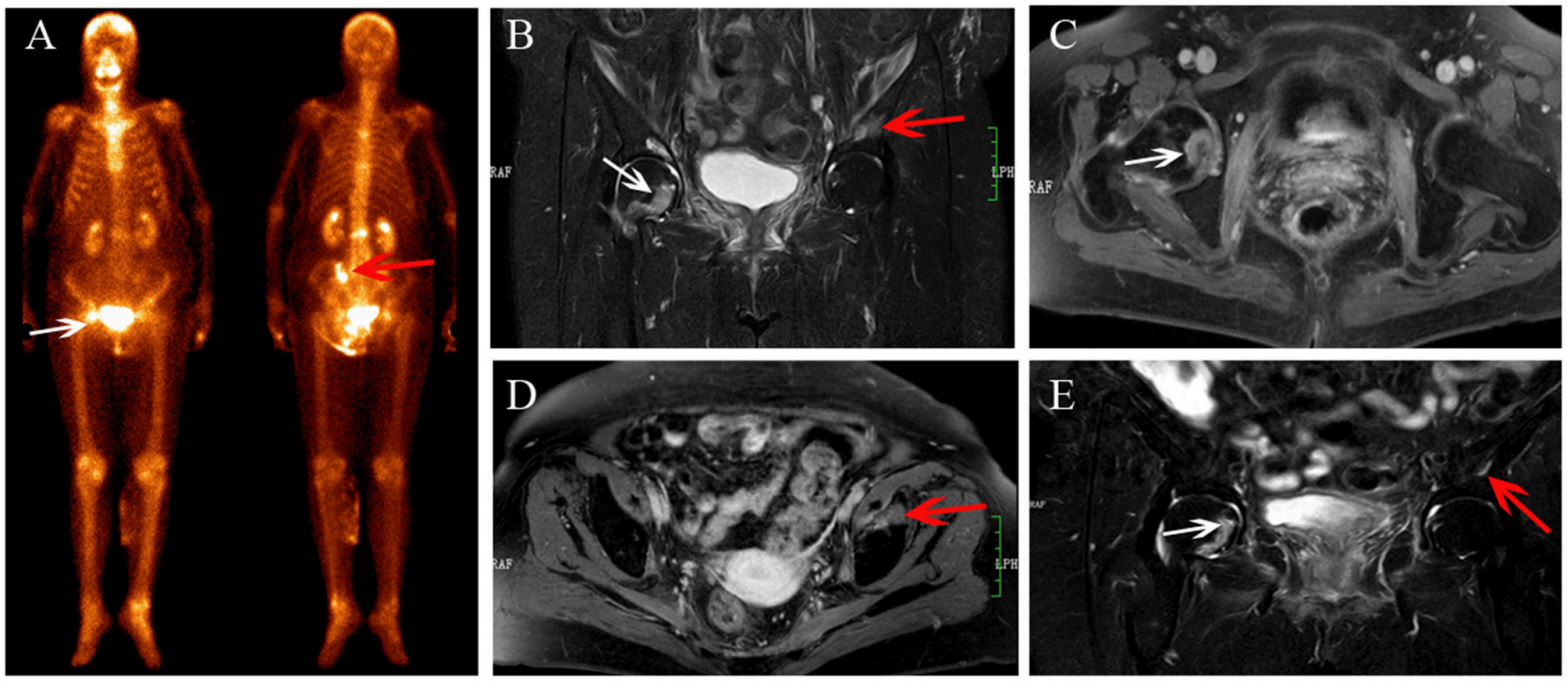
Multiple IFs after radiotherapy. BS **(A)** shows an increase in uptake in the right femoral head (white arrows) and the left ilium (red arrow). The IFs show hyperintensity on coronal FS T2-weighted **(B)** image, and right femoral head shows osteolytic change (white arrow). IFs show enhancement on contrast enhanced T1-weighted images **(C,D)**. At MRI follow-up after 12 months, the area of IFs has decreased **(E)**, and the fracture line appeared in the right femoral head (white arrow).

**Figure 4:**
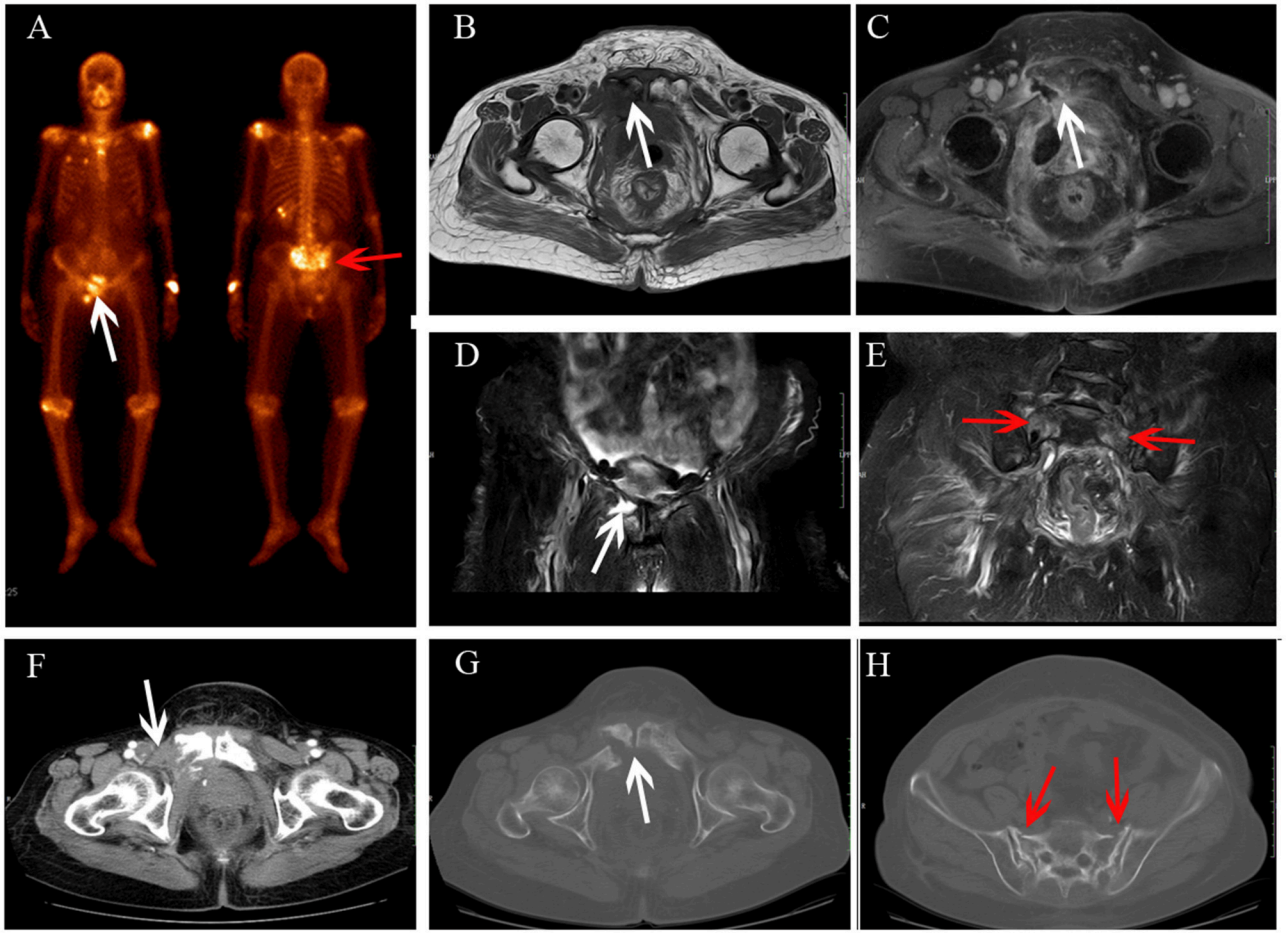
Sacrum IF combined with pubis metastasis. BS **(A)** shows the “Honda sign” (red arrow) and right pubis displays an increase in uptake (white arrow). The right pubis shows soft tissue change on axial T1-weighted **(B)** and enhanced T1-weighted image **(C)**, and osteolytic change on coronal FS T2-weighted image **(D)**. The sacrum shows hyperintensity on coronal FS T2-weighted image (**E**, red arrow). At CT follow-up after 6 months, the right pubis shows soft tissue tumor with osteolytic change **(F)** and pathological fracture **(G)**; the bilateral sacrum shows osteosclerosis with fracture lines **(H)**.

**Figure 5:**
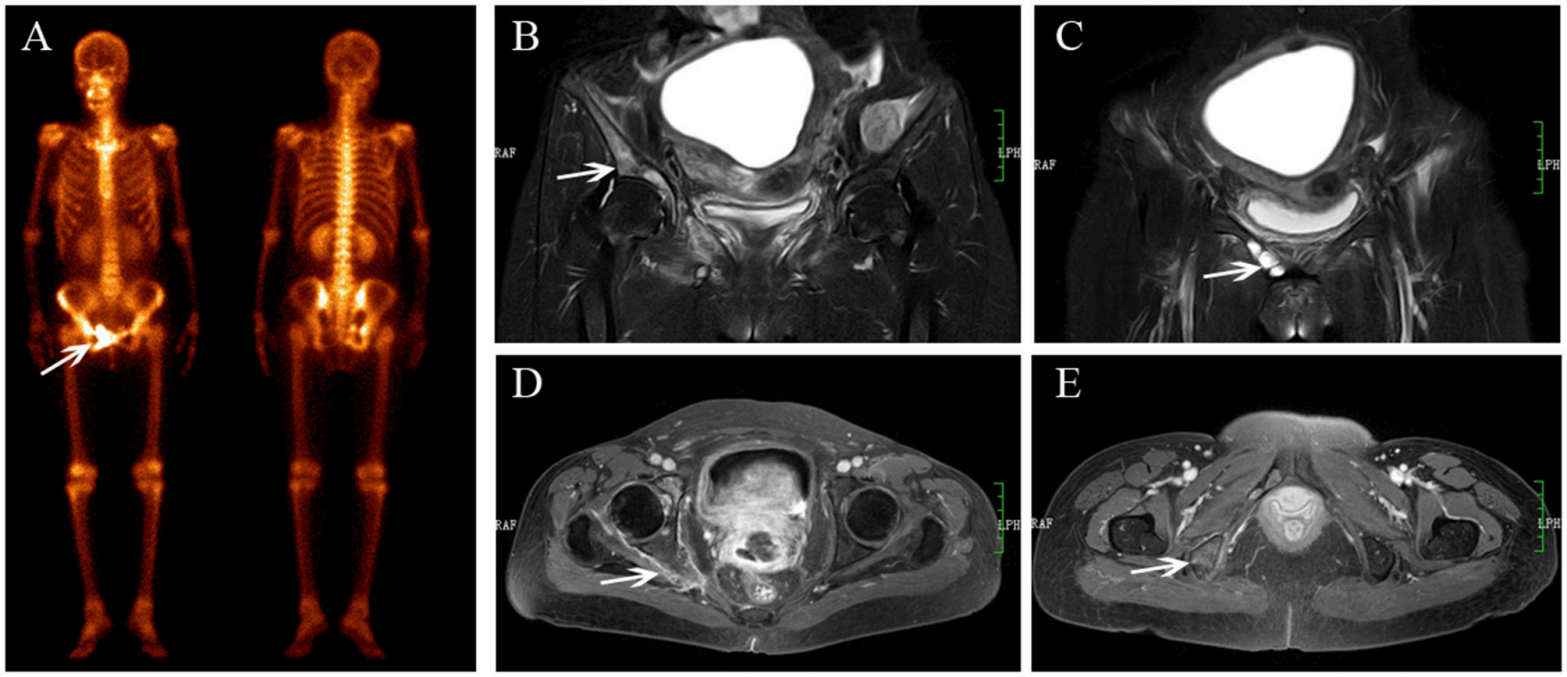
Multiple metastases after radiotherapy. BS **(A)** shows increasing uptake in the right acetabulum, pubis and ischium. The lesions show hyperintensity on coronal FS T2-weighted image **(B)**, osteolytic change (**C**, white arrow) and enhancement with soft tissue change **(D,E)**.

**Figure 6:**
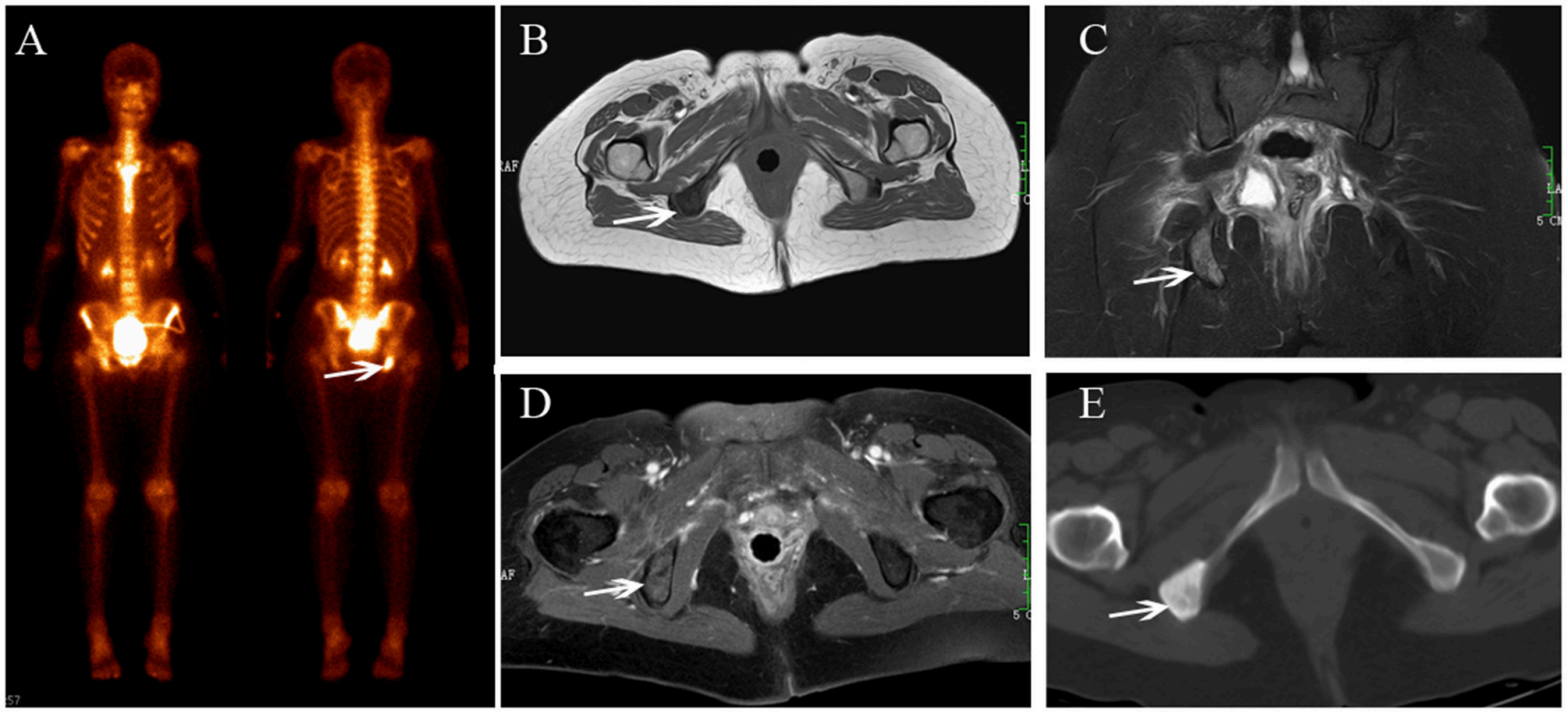
Ischium metastasis after radiotherapy. BS **(A)** shows increasing uptake in the right ischium. The lesion shows hypointensity on axial T1-weighted **(B)**, hyperintensity on coronal FS T2-weighted image **(C)**, and enhancement on enhanced T1-weighted images **(D)**. CT shows osteogenic change **(E)**.

Of the 40 patients, the median interval time from RT to lesion detection by BS was 11 months (range 5–35 months). All patients have been followed by MRI for further diagnosis, and the median interval time from BS to MRI evaluation was 6 days (range 2–13 days). The median follow-up period was 18 months (range 12–30 months) after MRI. Based on the gold standard, 30/40 (75%) patients were diagnosed with IFs (Figures 1–3) and 10 patients (25%) were diagnosed with bone metastases (Figures 4–6). Two of the 10 patients with bone metastases also had IF lesions (Figure 4). Of these 32 patients involved in IFs, 11 patients (34.4%) were developed with multiple fracture sites (Figures 2, 3), and 20 patients (62.5%) with sacral IFs showed a variant of “H-sign” on the BS (Figures 1, 2, 4). Of the 10 patients involved in bone metastases, three patients (33.3%) were developed with multiple metastases (Figure 5), and only one patient had both bone metastases and sacral IF showed a “H-sign” on the BS (Figure 4).

### Sites of IFs and Bone Metastases

According to the gold standard, a total of 57 lesions were included in our analysis; 43 lesions were IFs and 14 were bone metastases. The diagnosis and location of the 57 lesions are summarized in Table 2. IFs were most frequently observed in the sacrum, accounting for 67.4% (29/43) of the lesions. Other lesion locations included acetabulum (4 sites), pubis (3 sites), femoral head (2 sites), ischium (2 sites), L5 vertebra (1site), femoral neck (1 site), and iliac wing (1 site). Bone metastases were located at the iliac wing (4 site), sacrum (3 sites), acetabulum (2 sites), L5 vertebra (2 sites), femoral head (1 site), pubis (1 site), and ischium (1 site).

**Table 2:** Final diagnosis and location of the 57 bone lesions.

**Locations**	**IF**	**Bone metastasis**	**Total**
Sacrum	29	3	32
Acetabulum	4	2	6
Pubic ramus	3	1	4
Femoral head	2	1	3
Ischium	2	1	3
Femoral neck	1	0	1
Iliac wing	1	4	5
L5 vertebra	1	2	3
Total	43	14	57

### MRI Diagnostic Efficacy in the Identification of IF and Bone Metastasis

Based on the reference, the 57 lesions detected by BS, which were also found using MRI by both observers. Of the 43 IFs, reactive bone marrow edema was seen in 100% of lesions and enhancement was seen in 81.4% (35/43) of lesions by both observers (Figures 1–3). Observer 1 noted fracture lines in 83.7% (36/43) of IFs, and observer 2 showed that 79.7% (34/43) of IFs had fracture lines (Figures 1, 2). Two lesions showed that osteolytic change without fracture lines were underdiagnosed (Figure 3), including one acetabulum IF and one femoral head IF. For the assessment of bone metastasis, osteolytic destruction with soft tissue mass was observed in 13/14 (92.86%) lesions for both observers (Figures 4, 5); one lesion in the ischium only showed that osteogenic change was underdiagnosed (Figure 6).

Based on the mean recorded scores of the two observers, the sensitivity, specificity and accuracy for diagnosing IFs were 95.3% (41/43), 92.8% (13/14), and 94.7% (54/57), respectively. The interobserver variability was determined to be very good (*k* = 0.962).

## Discussion

Both RT-induced IF and bone metastasis show an increased uptake on a BS after radiotherapy, and the typical “H sign” of IF does not always appear, which can be misinterpreted as bone metastasis. However, MRI has the ability to detect IF by bone marrow edema pattern and fracture lines. In the present study, we found that MRI generated a high sensitivity and specificity for diagnosing RT-induced IFs and showed a good interobserver variability. MRI would be an important diagnostic technique when a lesion is equivocal to diagnose after a BS in cervical cancer patients with increased uptake after radiotherapy.

BS is a highly sensitive nuclear medicine procedure that is widely used to detect bone metastasis. However, trauma, inflammation, and malignant or benign bone diseases all exhibit increased uptake of the radiotracer, and BS shows low specificity (19, 20). For the diagnosis of IF, HS is known as a characteristic sign for sacral IF, with a positive predictive value of up to 94% (12). However, this sign usually disappears, as our study revealed that 60.6% of cases with IF showed “H-sign” on a BS, which was consistent with a previous meta-analysis result (21). When a “H-sign” is absent in a patient with known cancer, the diagnosis of IF cannot always be established by using BS alone, and it is difficult to differentiate IF from malignant bone lesions.

An accurate diagnosis of IF and differentiating it from bony metastasis is crucial, due to an erroneous diagnosis that could lead to patients undergoing biopsy or treated by inappropriate chemotherapy or RT (7, 22, 23). MRI is a useful and alternative tool, due to its high soft-tissue contrast, multiplanar imaging, and avoidance of ionizing radiation. As for the diagnosis of IF by MRI, reactive bone marrow edema and fracture lines in the areas of radiation have be identified as reliable features (9, 11, 14, 15, 22). Our study demonstrated that MRI resulted in a considerably high diagnostic efficacy in the distinction of RT-induced IF and bone metastasis, the overall accuracy was up to 94.7%, which supported an accuracy of 89.6% performed by SPE/CT (17). Furthermore, we found that the interobserver variability of MRI was excellent for the diagnosis of IF (*k* = 0.962).

Bone marrow edema shows hypointensity on T1-weighted images and hyperintensity on STIR or FS-T2W images. Fracture line shows a linear structure on all MR sequence. We found that the incidence rate of reactive bone marrow edema was 100%. This result was higher than previous studies have reported, which showed that 89–96.3% of cases demonstrated reactive bone marrow change on the MRI (9, 11). These differing results could be due to the fact that IFs involved in our study were all previously detected by BS, and the bone marrow edema pattern may be associated with the increased uptake on BS. Some foregoing studies have shown that MRI is limited in detecting fracture lines (24, 25). However, in our study, fracture lines were noted in approximately 83.7% of IF lesions, probably due to the fact that both fat suppression T2-weighted and contrast-enhanced T1-weighted imaging were performed in all cases, which showed more sensitivity to detect a low signal fracture line than conventional T1-weighted or T2-weighted imaging (9, 22, 25). However, not all IFs present reactive bone marrow edema and fracture lines on MRI, IF may occasionally show osteolytic change. In the present study, two lesions showed osteolytic change combined with enhancement were underdiagnosed. Therefore, osteolytic lesions may not completely exclude the diagnosis of IF.

Pelvic multiple lesions are frequently found in patients with cervical cancer after radiotherapy. It is quite significant to confirm whether there are any bone metastasis in these multiple lesions, which has a profound influence on the management of the patient with cervical cancer, and the presence of bone metastases indicates shortened patient survival time (26, 27). In our study, 13 of the 40 patients with multiple lesions and bone metastasis combined with IFs were found in two patients. Hematogenous bone metastasis is considered to start in the bone marrow, and the majority of bone metastases in cervical cancer seem to be of an osteolytic nature (28). We found that MRI could diagnose 92.8% (13/14) of bone metastasis and 7.1% (1/14) of lesions with osteogenic change was underdiagnosed by MRI. Hence, osteogenic lesions without soft tissue mass in cervical cancer could not completely exclude the diagnosis of bone metastasis, and conventional MRI could have some limitations in the identification of osteogenic bone metastasis from RT-induced IF due to the similar MRI findings.

There were several limitations of our study. First, this was a single-institution, retrospective study with 30 patients with IF and only 10 patients with bone metastases. Second, because only a small number of cases had diffusion-weighted imaging (DWI), we were unable to explore potential diagnostic value of DWI and apparent diffusion coefficient (ADC), which has proven to be a very useful technique for differentiating benign and malignant bone marrow disease (29). Third, none of the IF lesions and bone metastases were diagnosed based on histopathology because that would have required either surgery or puncture, which was generally impractical and unnecessary. Finally, MRI results are involved in the reference standard, which is later used to evaluate the performance of MRI, may potentially introduce significant bias, affecting the reliability of the results.

In conclusion, increased uptake on BS in the radiation field is a common finding during follow-up in patients with cervical cancer that underwent pelvic RT. Differential diagnosis should be performed when the pelvis shows emerging lesions on BS after radiotherapy in patients with cervical cancer. MRI is an useful diagnostic technique for the further characterization of these emerging lesions detected by BS, MRI shows great diagnostic efficiency in the differentiation of IF and bone metastasis.

## Data Availability

The authors state that datasets generated for this study included some example images and some compounded clinical information, but this is hardly a complete data set.

## Ethics Statement

The ethics of this study were reviewed and approved by the Institutional Review Board of Affiliated Cancer Hospital and Institute of Guangzhou Medical University. This was a retrospective study, which used clinical data acquired from cervical cancer patients.

## Author Contributions

XZ, JZ, and RT: conception and design; XZ, JL, and JZ: manuscript writing; LZ, JY, and JZ: provision of study materials or patients; JY and ZC: collection and assembly of data; JL and BL: MRI analysis and interpretation; LZ: BS image analysis and interpretation; XZ and JZ: statistical analysis; JZ and RT: final approval of manuscript.

### Conflict of Interest Statement

The authors declare that the research was conducted in the absence of any commercial or financial relationships that could be construed as a potential conflict of interest.
